# Acute Arterial Ischemia Secondary to Intrapelvic Acetabular Migration: A Multidisciplinary Approach

**DOI:** 10.7759/cureus.75231

**Published:** 2024-12-06

**Authors:** Carlo A Sánchez, Monica Leon, Andrea Falconi, Alex D Contreras, Andrés Dryjanski, Claudia A Martínez

**Affiliations:** 1 Vascular Surgery, Centro Médico Nacional 20 de Noviembre, Mexico City, MEX; 2 Faculty of Health Science, Universidad Anahuac Mexico Norte, Huixquilucan, MEX; 3 Orthopaedics and Trauma, Hospital Español de México, Mexico City, MEX

**Keywords:** acetabular migration, acute limb ischemia, arterial ischemia, hip disarticulation, limb salvage, peripheral arterial disease, prosthetic joint infection, thrombosis

## Abstract

This case report evaluates current diagnostic and treatment approaches for intrapelvic acetabular migration, focusing on the rare but serious complication of acute limb ischemia following hip arthroplasty. A 67-year-old female with a history of total hip arthroplasty 10 years ago presented with acute limb ischemia after experiencing a traumatic event 72 hours prior, which had caused displacement of her hip prosthesis. Notably, she had a history of a traumatic event two years earlier for which she had been advised to undergo surgical correction, which she had refused. A multidisciplinary team assessed her preoperatively. She was diagnosed with SVS III irreversible acute limb ischemia due to compression of the external iliac artery from the prosthesis migration, prompting an emergency hip disarticulation. The patient successfully underwent hip disarticulation and mechanical thrombectomy of the external iliac artery using a Fogarty catheter. Postoperative recovery was notable, with significant pain relief, improved mental status, and restoration of the iliac pulse.

Early diagnosis and management of acute arterial injury are crucial to preventing severe outcomes. This report highlights the importance of timely intervention to mitigate limb-threatening and life-threatening complications. It underscores the need for vigilant monitoring during hip replacements and the effectiveness of a multidisciplinary approach in complex cases. Continued research is essential to enhance diagnostic and therapeutic strategies for this rare yet critical complication and to improve overall patient outcomes.

## Introduction

Total hip arthroplasty is one of the most effective orthopedic procedures; however, its proximity to major blood vessels makes vascular injury a potentially lethal complication. Recent literature has highlighted vascular injuries associated with acetabular fractures as rare occurrences, but even rarer is the involvement of the external iliac artery, with reported mortality rates ranging from 75% to 83% [1-2].

The classification of arterial ischemia is critical for assessing the severity of these injuries, particularly the Society for Vascular Surgery (SVS) grading system. In this system, SVS III indicates irreversible damage, which underscores the significance of timely intervention. The physiopathology of ischemia involves a reduction in blood flow, leading to tissue hypoxia and cellular necrosis. This progression emphasizes the importance of early recognition and treatment, as the absence of timely intervention can result in devastating consequences such as irreversible tissue damage, organ failure, limb amputation, and increased morbidity or mortality.

Misdiagnosing vascular injuries can significantly exacerbate these outcomes. The varied clinical presentations of such injuries often pose diagnostic challenges, highlighting the need for healthcare professionals to remain vigilant in recognizing these complications to facilitate prompt treatment. We describe a case of a patient with a hip replacement who experienced a traumatic event resulting in hip prosthesis displacement and subsequent acute lower limb ischemia classified as SVS III. The patient was treated with hip disarticulation and thrombectomy of the external iliac artery [3-4].

## Case presentation

The patient was a 67-year-old female who had been referred to our emergency department due to pain and ischemic skin changes in the left lower limb, suggestive of prior vascular compromise. She had been treated 48 hours earlier at another institution with analgesics but came to our emergency department hemodynamically stable yet exhibiting altered consciousness, lethargy, and newly developed skin ulcerations. On physical examination, the limb was cold with ischemic discoloration and darkened ulcerations on the posterior thigh. Neurovascular assessment revealed absent femoral and distal pulses, loss of sensation from the infragenicular area to the foot, and complete immobility of the extremity.

**Figure 1:**
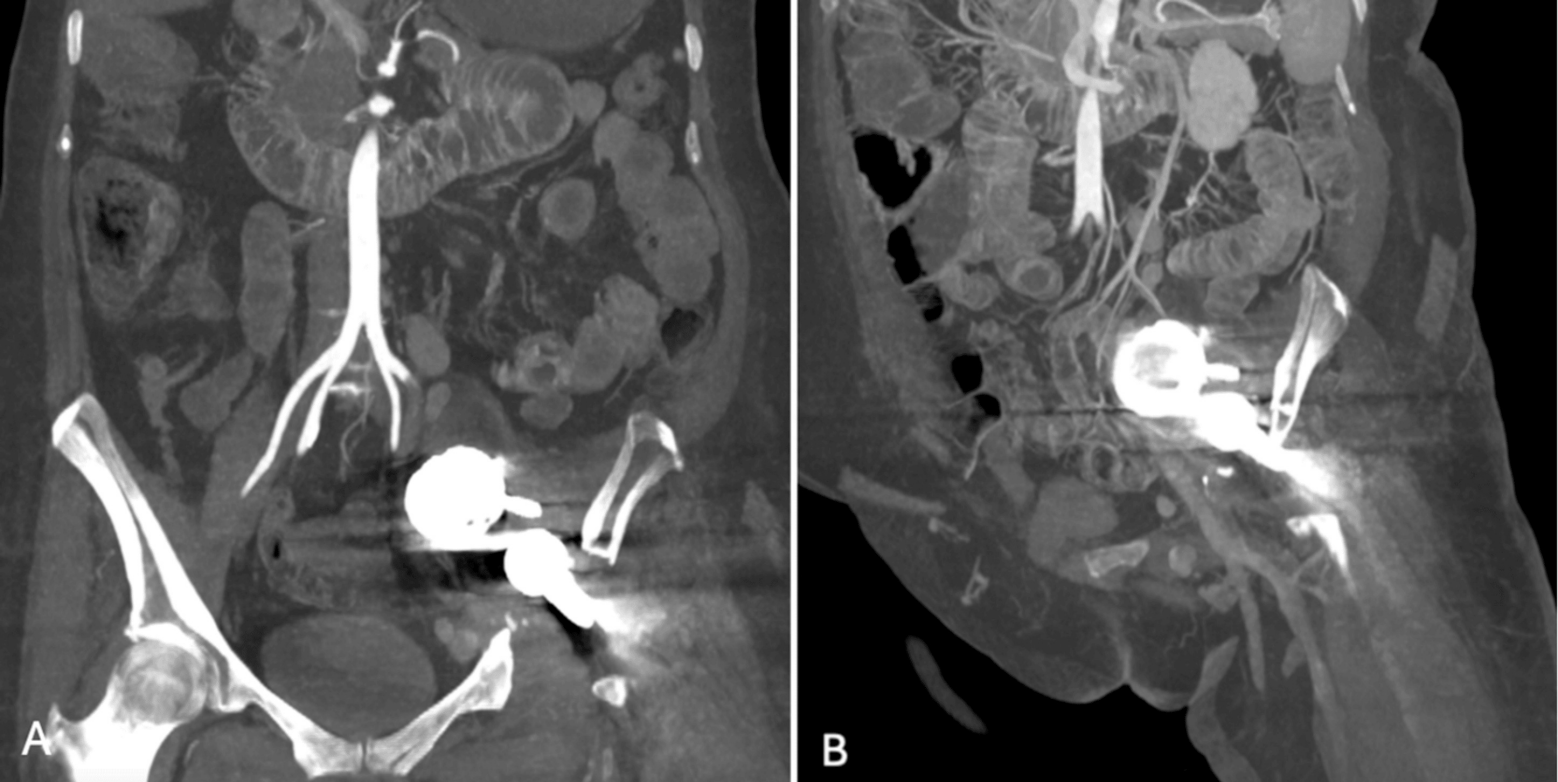
Angiotomography images A) Hip prosthesis intraabdominal migration, with disruption of the Kohler line, severe bone loss, and disruption of the ischial rim. B) Sagittal plane

The patient had undergone a total hip arthroplasty 10 years earlier and experienced a traumatic event two years prior for which she had been advised to undergo surgical correction, which she had declined. Complementary arteriography demonstrated acetabular detachment with posterior and medial migration of the hip prosthesis beyond the iliopectineal line (Figure 1).

**Figure 2:**
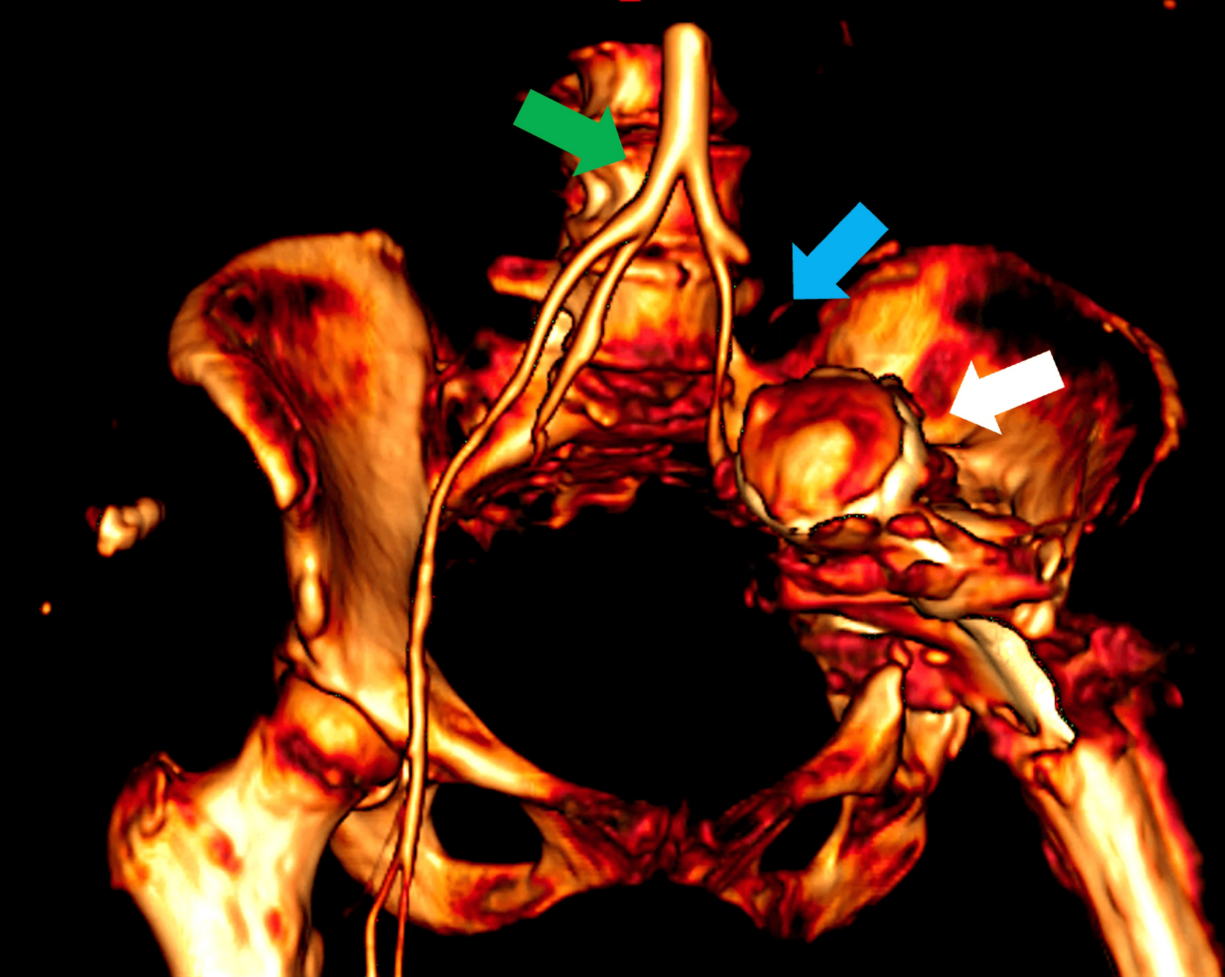
Angiotomography 3D reconstruction Green arrow: aorto-iliac bifurcation. Blue arrow: thrombosis of the left external iliac artery is seen, without contrast towards the distal segment. White arrow: left acetabular migration

Vascular reconstructions revealed external iliac artery compression with loss of distal vascular irrigation (Figure 2). 

A diagnosis of acute thrombosis of the external iliac artery was made, classified as SVS III for the left lower limb, prompting an open emergency surgical treatment. Exposure of the left femoral vessels was performed through an infrainguinal traditional incision, followed by successful proximal thrombectomy, using a Fogarty 4Fr catheter, of the external iliac artery, resulting in the removal of acute thrombus. During the procedure, liquefaction and ischemic subcutaneous cellular tissue were observed up to the proximal femoral portion, leading to the decision to perform disarticulation. The initial incision was extended circumferentially around the limb, and disarticulation was completed using Boyd's surgical technique. This technique involves a racket-shaped incision in the thigh and groin, with muscles detached and reflected, vessels secured and divided, and gluteal muscles, iliopsoas, and obturator externus forming a weight-bearing flap. A systematic dissection was performed, avoiding weight-bearing on suture lines to ensure viable muscle flaps [5].

**Figure 3:**
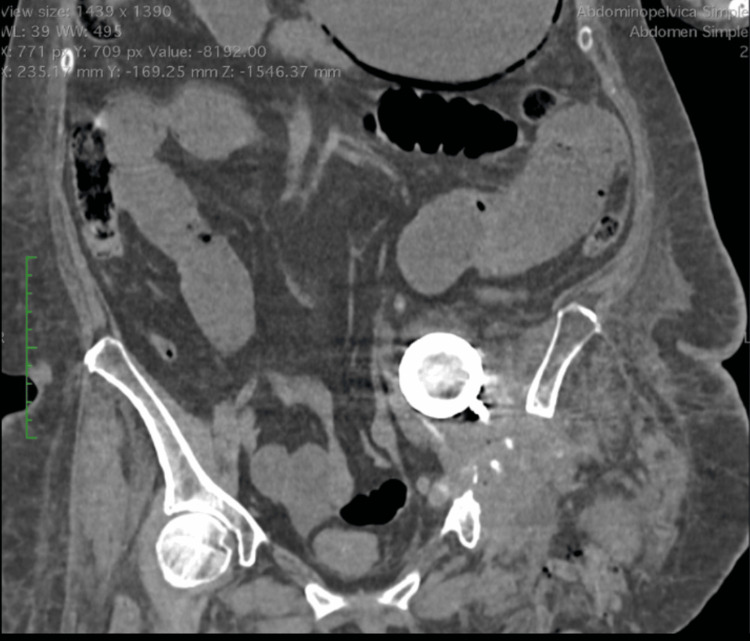
Tomography findings Postoperative control study, with the acetabular component of the hip prosthesis still intra-abdominally

During the dissection, tissue adjacent to the femoral portion of the prosthesis showed changes consistent with melanosis. Due to the patient's hemodynamic status and the challenging exposure of the acetabular portion in the abdomen, it was decided to complete the disarticulation by removing the entire limb and femoral portion of the prosthesis, adopting the abdominal approach for the removal of the acetabular portion for a second surgical stage (Figure 3).

Laboratory findings revealed significant abnormalities consistent with acute limb ischemia and reperfusion injury. Creatine phosphokinase (CPK) was markedly elevated at 5469 U/L, indicating severe muscle injury. Renal function tests showed an elevated blood urea nitrogen (BUN) of 51 mg/dL and a creatinine level of 1.55 mg/dL, with a BUN/creatinine ratio of 32, suggesting acute kidney impairment. The hepatic function tests indicated mild transaminitis, with aspartate aminotransferase (AST) at 81 U/L.

The complete blood count revealed leukocytosis, with leukocytes at 20.04 K/µL and an elevated neutrophil count of 17.85 K/µL. Albumin was low at 2.6 g/dL, indicating a state of hypoalbuminemia. Coagulation studies showed D-dimer at 3.8 µg/mL, elevated fibrinogen at 579.9 mg/dL, and a sedimentation rate (VSG) of 46 mm/h, suggesting an inflammatory process. Procalcitonin was elevated at 2.22 ng/mL, indicating a potential infectious or inflammatory response (Table 1).

**Table 1:** Postoperative laboratory findings AST: aspartate aminotransferase; BUN: blood urea nitrogen; CPK: creatine phosphokinase

Parameter	Patient value	Reference range	
CPK	5469 U/L	20-200 U/L	
BUN	51 mg/dL	7-20 mg/dL	
Creatinine	1.55 mg/dL	0.6-1.3 mg/dL	
BUN/creatinine Ratio	32	10-20	
AST	81 U/L	8-40 U/L	
Leukocytes	20.04 K/µL	4.0-11.0 K/µL	
Neutrophils	17.85 K/µL	2.0-7.0 K/µL	
Albumin	2.6 g/dL	3.5-5.0 g/dL	
D-dimer	3.8 µg/mL	<0.5 µg/mL	
Fibrinogen	579.9 mg/dL	200-400 mg/dL	
Sedimentation rate (VSG)	46 mm/h	0-20 mm/h	
Procalcitonin	2.22 ng/mL	<0.1 ng/mL	

The patient remained in the hospital for a total of three days, with two days spent in the surgical ICU. Postoperative complications included ischemia-reperfusion injury that occurred too after the procedure.

## Discussion

Intrapelvic acetabular migration is a rare and severe complication of hip arthroplasty, posing significant risks to neurovascular structures and pelvic organs, and potentially leading to lethal complications. Despite its low prevalence, the frequency of this condition has been increasing in recent years, attributed to rising life expectancy and the growing number of hip prosthesis implants [2]. Causes of intrapelvic migration include mechanical factors, malposition leading to chronic instability, and traumatic events. Although the involvement of the iliac artery is rare, even rarer is the thrombosis of the external iliac artery due to hip prosthesis intrapelvic migration [6,7]. In our patient, we concluded that it was a combination of a traumatic event and potential underlying low bone density disease that led to the protrusion of the femoral head [7,8]. In this context, vascular lesions can arise from various etiologies, with the thrombosis of the external iliac artery likely caused by external compression resulting in significant wall stress [8].

Previous literature has documented the diagnosis of vascular injuries primarily through angiography or angiotomography, particularly in cases of traumatic pelvic fractures or intra-surgical complications related to hip replacement [2-6]. The prognosis remains closely linked to the time of evolution, with a high risk of limb and life-threatening outcomes. Previous case reports have emphasized the critical importance of early diagnosis and timely intervention to avoid extremity loss and long-term complications [9]. This case underlines the necessity for heightened awareness and suspicion among clinicians regarding vascular injury following hip arthroplasty. Early identification can significantly alter the course of treatment and outcomes for patients. Studies suggest that timely revascularization procedures can improve limb salvage rates, particularly when intervention occurs before irreversible ischemic changes set in. In cases where vascular compromise is suspected, immediate imaging and intervention should be prioritized to facilitate timely decision-making.

In discussing the complexities of treatment options, the decision between revascularization and amputation is not straightforward. While revascularization is often the preferred initial approach, especially in cases of acute ischemia, it must be balanced against the potential for irreversible damage, particularly in class III ischemia. Previous studies indicate that outcomes of revascularization can vary widely based on the extent of tissue ischemia and the time elapsed before surgical intervention. Our ultimate goal was to provide the patient with the opportunity for healing, despite the inherent high risks of reperfusion damage and associated complications. We believed that a successful revascularization could allow the wound to heal, thus improving the patient’s quality of life. In cases where revascularization is deemed unlikely to restore sufficient function, amputation may ultimately be necessary, which underscores the need for a multidisciplinary approach involving vascular surgeons, orthopedic surgeons, and rehabilitation specialists to determine the best course of action for the patient.

Due to the life-threatening nature of the outcomes associated with intrapelvic acetabular migration, surgeons must maintain a high index of suspicion for early vascular injury. Prompt diagnosis and intervention not only improve limb salvage rates but also significantly enhance overall patient prognosis, emphasizing the need for ongoing education and awareness within the surgical community.

## Conclusions

Early diagnosis and timely intervention are critical in managing acute limb ischemia to prevent fatal outcomes, necessitating a multidisciplinary approach. In our case, despite the patient’s evolution exceeding 72 hours, hip disarticulation combined with embolectomy of the external iliac artery was determined to be the most effective treatment for addressing the irreversible ischemic episode and mitigating the systemic inflammatory response. Each case of acute limb ischemia should be evaluated individually, taking into account the patient's specific clinical circumstances, risk factors, and potential for recovery. The importance of early detection of vascular injury cannot be overstated, as prompt intervention can significantly alter the course of treatment and improve patient outcomes.

Current and future directions in the management of acute limb ischemia must focus on advancements in diagnostic imaging and surgical techniques. Continuous research into optimizing revascularization strategies and understanding the complexities of reperfusion damage will play a crucial role in enhancing recovery rates and reducing complications. Prioritizing early clinical detection and individualized treatment plans will help us better navigate the challenges associated with acute limb ischemia, ultimately improving patient care and outcomes in these complex cases.
